# Supplementary material for: Impact of COVID-19 pandemic on physician-scientist trainees to faculty one year into the pandemic
Source: BMC Med Educ. 2024 May 28;24:587. doi: 10.1186/s12909-024-05541-9 (PMC11134762; doi:10.1186/s12909-024-05541-9)
Supplement: Supplementary file 3 — Supplementary Material 3. [file 12909_2024_5541_MOESM3_ESM.ppt]

## Slide 1
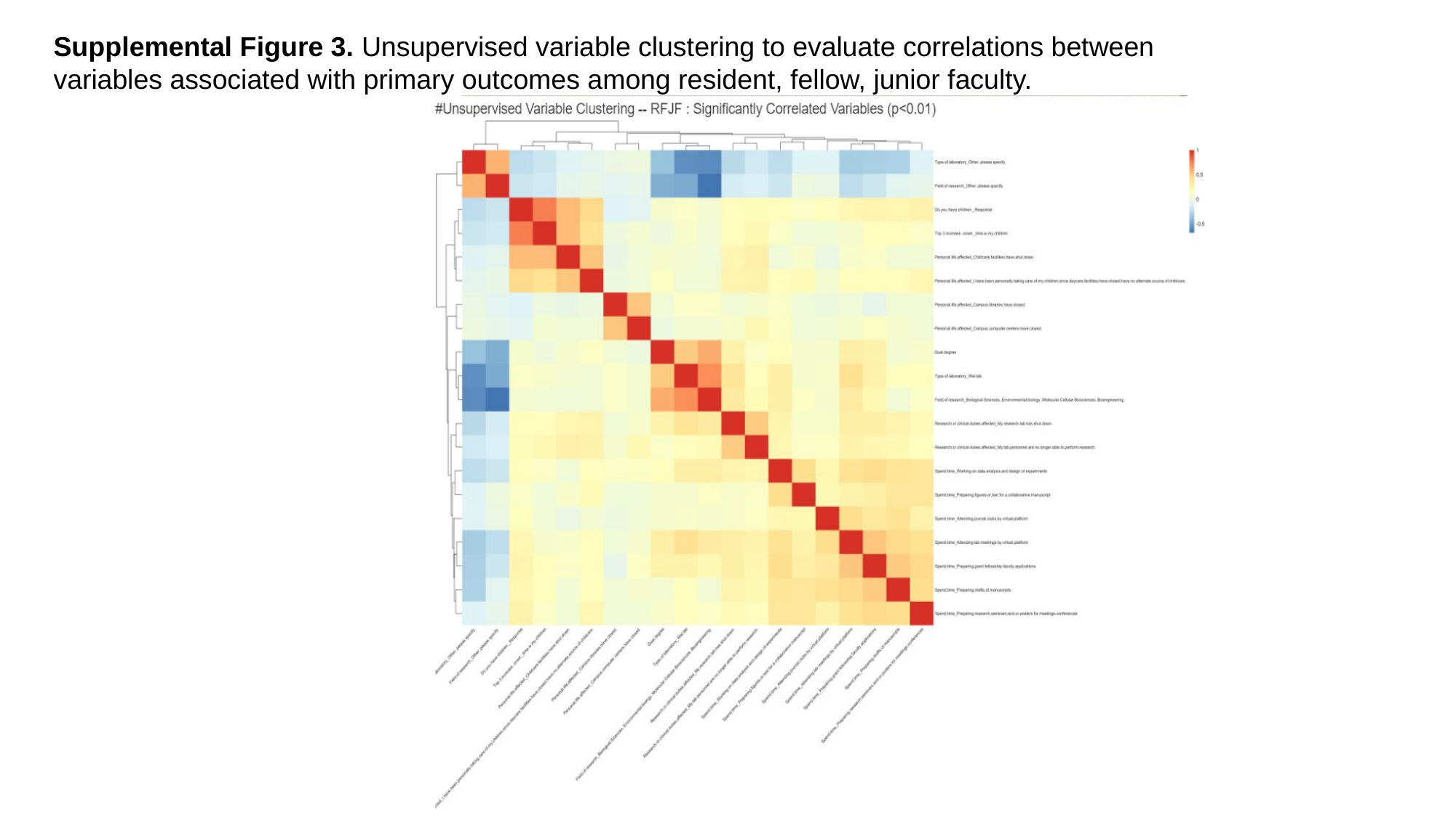

Supplemental Figure 3. Unsupervised variable clustering to evaluate correlations between variables associated with primary outcomes among resident, fellow, junior faculty.
