# Supplementary material for: Impact of COVID-19 pandemic on physician-scientist trainees to faculty one year into the pandemic
Source: BMC Med Educ. 2024 May 28;24:587. doi: 10.1186/s12909-024-05541-9 (PMC11134762; doi:10.1186/s12909-024-05541-9)
Supplement: Supplementary file 4 — Supplementary Material 4. [file 12909_2024_5541_MOESM4_ESM.docx]

| **Medical students** | |  | |  |
| --- | --- | --- | --- | --- |
|  | Total (N=179) | | Summary | |
| Age | Mean (SD) | | 26 03 (3 32) | |
|  | Median (IQR) | | 25 (23, 28) | |
|  | Range | | (21, 37) | |
| Gender | Female | | 96 (54%) | |
|  | Male/Other | | 83 (46%) | |
| Do you identify as transgender? | No | | 179 (100%) | |
| American Indian or Alaskan Native | No | | 175 (98%) | |
|  | Yes | | 4 (2%) | |
| Black or African American | No | | 168 (94%) | |
|  | Yes | | 11 (6%) | |
| Chinese | No | | 161 (90%) | |
|  | Yes | | 18 (10%) | |
| Vietnamese | No | | 176 (98%) | |
|  | Yes | | 3 (2%) | |
| Korean | No | | 170 (95%) | |
|  | Yes | | 9 (5%) | |
| Japanese | No | | 178 (99%) | |
|  | Yes | | 1 (1%) | |
| Asian Indian | No | | 172 (96%) | |
|  | Yes | | 7 (4%) | |
| Filipino | No | | 177 (99%) | |
|  | Yes | | 2 (1%) | |
| Other Asian | No | | 175 (98%) | |
|  | Yes | | 4 (2%) | |
| Native Hawaiian or Other Pacific Islander | No | | 179 (100%) | |
| White | No | | 64 (36%) | |
|  | Yes | | 115 (64%) | |
| Multi-racial | No | | 160 (89%) | |
|  | Yes | | 19 (11%) | |
| Ethnicity | Hispanic or Latino/a | | 19 (11%) | |
|  | Not/other | | 160 (89%) | |
| Are you married or partnered | No | | 110 (61%) | |
|  | Yes | | 66 (37%) | |
| Do you have children | No | | 165 (92%) | |
|  | Yes | | 14 (8%) | |
| Region | Midwest | | 47 (26%) | |
|  | Northeast | | 76 (42%) | |
|  | Northwest/Southwest | | 13 (7%) | |
|  | South/Southeast | | 42 (23%) | |
| Is your institution public or private | Private | | 102 (57%) | |
|  | Public | | 77 (43%) | |
| Dual degree | No | | 14 (8%) | |
|  | Yes | | 165 (92%) | |
| Have you received a COVID 19 vaccination | No | | 1 (1%) | |
|  | Yes | | 178 (99%) | |
| Training year | Medical School-Year 1 | | 69 (39%) | |
|  | Medical School-Year 2 | | 54 (30%) | |
|  | Medical School-Year 3 | | 18 (10%) | |
|  | Medical School-Year 4 | | 38 (21%) | |
| Medical training affected Delay in or cancellation of lectures | No | | 127 (71%) | |
|  | Yes | | 52 (29%) | |
| Medical training affected Lectures have been changed to remote access only/virtual platforms | No | | 38 (21%) | |
|  | Yes | | 141 (79%) | |
| Delay in or cancellation of medical education meetings or advisor meetings | No | | 137 (77%) | |
|  | Yes | | 42 (23%) | |
| Medical education or advisor meetings have been made virtual | No | | 50 (28%) | |
|  | Yes | | 129 (72%) | |
| Delay in or cancellation of clerkship rotations | No | | 144 (80%) | |
|  | Yes | | 35 (20%) | |
| Virtual clinical rotations have been implemented | No | | 151 (84%) | |
|  | Yes | | 28 (16%) | |
| Removal from clinical clerkship rotations | No | | 166 (93%) | |
|  | Yes | | 13 (7%) | |
| Delay in or cancellation of medical tests/shelf exams | No | | 166 (93%) | |
|  | Yes | | 13 (7%) | |
| Delay in or cancellation of standardized tests | No | | 154 (86%) | |
|  | Yes | | 25 (14%) | |
| Difficulty in accomplishing on-boarding or administrative tasks in preparation for residency | No | | 168 (94%) | |
|  | Yes | | 11 (6%) | |
| Given option for early graduation | No | | 179 (100%) | |
| Graduation has been delayed | No | | 175 (98%) | |
|  | Yes | | 4 (2%) | |
| Proportion of time spending on Childcare/homeschooling | >0 | | 16 (12%) | |
|  | 0 | | 122 (88%) | |
| Proportion of time spending on Clinical duties | <=45% | | 102 (57%) | |
|  | >45% | | 77 (43%) | |
| Proportion of time spending on Administrative duties | <=45% | | 145 (81%) | |
|  | >45% | | 34 (19%) | |
| Proportion of time spending on Research/scholarly activities | <=45% | | 102 (57%) | |
|  | >45% | | 77 (43%) | |
| Proportion of time spending on Personal time | <=45% | | 163 (91%) | |
|  | >45% | | 16 (9%) | |
| Proportion of time spending on Volunteer activities | <=45% | | 164 (92%) | |
|  | >45% | | 15 (8%) | |
| Personal life affected by campus transportation or local transit being shut down | No | | 158 (88%) | |
|  | Yes | | 21 (12%) | |
| Personal life affected by childcare facilities being shut down | No | | 170 (95%) | |
|  | Yes | | 9 (5%) | |
| I have been personally taking care of my children since daycare facilities have closed/have no alternate source of childcare | No | | 175 (98%) | |
|  | Yes | | 4 (2%) | |
| Personal life affected by homeschooling my children | No | | 178 (99%) | |
|  | Yes | | 1 (1%) | |
| Personal life affected by taking care of elderly loved ones | No | | 178 (99%) | |
|  | Yes | | 1 (1%) | |
| Personal life affected by campus libraries closures | No | | 106 (59%) | |
|  | Yes | | 73 (41%) | |
| Personal life affected by campus computer center closures | No | | 146 (82%) | |
|  | Yes | | 33 (18%) | |
| Personal life affected by working from home | No | | 83 (46%) | |
|  | Yes | | 96 (54%) | |
| I have lost my job due to the pandemic | No | | 178 (99%) | |
|  | Yes | | 1 (1%) | |
| I have been physically isolated from friends/family due to my work | No | | 80 (45%) | |
|  | Yes | | 99 (55%) | |
| I have still been able to keep in touch with friends/family via virtual platforms | No | | 53 (30%) | |
|  | Yes | | 126 (70%) | |
| I am living by myself thus am not concerned about quarantining from family members/roommates/partner | No | | 136 (76%) | |
|  | Yes | | 43 (24%) | |
| I have to self-quarantine away from my family/roommates/partner due to being exposed to COVID-19 or because of COVID-19-related symptoms (without confirmatory COVID-19 testing) | No | | 158 (88%) | |
|  | Yes | | 21 (12%) | |
| I have been exposed to COVID-19 or have symptoms (without confirmatory COVID-19 testing), but have no option to live in a different residence (hotel, institution facility) | No | | 162 (91%) | |
|  | Yes | | 17 (9%) | |
| I have been exposed to COVID-19 or have symptoms (without confirmatory COVID-19 testing), and have been offered an alternative residence (hotel, institution facility) | No | | 176 (98%) | |
|  | Yes | | 3 (2%) | |
| I have to self-quarantine away from my family/roommates/partner due to being exposed to COVID-19 and have tested positive for COVID-19 | No | | 176 (98%) | |
|  | Yes | | 3 (2%) | |
| I am currently being treated for symptoms/sequelae of being infected with COVID-19 | No | | 177 (99%) | |
|  | Yes | | 2 (1%) | |
| I have accrued significant medical debt after being treated for symptoms/sequelae of being infected with COVID-19 | No | | 179 (100%) | |
| My family/roommates/partner have been affected or infected because of my COVID-19 infection | No | | 165 (92%) | |
|  | Yes | | 14 (8%) | |
| The pandemic has had no effect on my personal life | No | | 165 (92%) | |
|  | Yes | | 14 (8%) | |
| Top 3 increase (onset) career success | No | | 125 (70%) | |
|  | Yes | | 54 (30%) | |
| Top 3 increase (onset) research | No | | 141 (79%) | |
|  | Yes | | 38 (21%) | |
| Top 3 increase (onset) friends/family | No | | 55 (31%) | |
|  | Yes | | 124 (69%) | |
| Top 3 increase (onset) time w/my children | No | | 166 (93%) | |
|  | Yes | | 13 (7%) | |
| Top 3 increase (onset) time w/my partner | No | | 110 (61%) | |
|  | Yes | | 69 (39%) | |
| Top 3 increase (onset) personal health | No | | 56 (31%) | |
|  | Yes | | 123 (69%) | |
| Top 3 increase (onset) exercise | No | | 106 (59%) | |
|  | Yes | | 73 (41%) | |
| Top 3 increase (onset) spending more time with hobbies | No | | 117 (65%) | |
|  | Yes | | 62 (35%) | |
| Top 3 increase (onset) No change | No | | 173 (97%) | |
|  | Yes | | 6 (3%) | |
| Working on data analysis and design of experiments | No | | 97 (54%) | |
|  | Yes | | 82 (46%) | |
| Reading scientific literature | No | | 56 (31%) | |
|  | Yes | | 123 (69%) | |
| Attending journal clubs by virtual platform | No | | 105 (59%) | |
|  | Yes | | 74 (41%) | |
| Attending lab meetings by virtual platform | No | | 96 (54%) | |
|  | Yes | | 83 (46%) | |
| Preparing drafts of manuscripts | No | | 112 (63%) | |
|  | Yes | | 67 (37%) | |
| Preparing grant/fellowship/faculty applications | No | | 168 (94%) | |
|  | Yes | | 11 (6%) | |
| Preparing dissertation chapters | No | | 160 (89%) | |
|  | Yes | | 19 (11%) | |
| Preparing review articles | No | | 160 (89%) | |
|  | Yes | | 19 (11%) | |
| Preparing research seminars and/or posters for meetings/conferences | No | | 158 (88%) | |
|  | Yes | | 21 (12%) | |
| Taking online courses to enhance skills | No | | 142 (79%) | |
|  | Yes | | 37 (21%) | |
| Performing computational modeling | No | | 163 (91%) | |
|  | Yes | | 16 (9%) | |
| Search/analysis of genomic/proteomic sequence data | No | | 166 (93%) | |
|  | Yes | | 13 (7%) | |
| Secondary data analysis | No | | 146 (82%) | |
|  | Yes | | 33 (18%) | |
| Working collaboratively to outline an experimental plan for a study | No | | 158 (88%) | |
|  | Yes | | 21 (12%) | |
| Preparing figures or text for a collaborative manuscript | No | | 138 (77%) | |
|  | Yes | | 41 (23%) | |
| Enhancing career development through institutional/online resources | No | | 144 (80%) | |
|  | Yes | | 35 (20%) | |
| Taking practice questions, reviewing board material for exams | No | | 103 (58%) | |
|  | Yes | | 76 (42%) | |
| Writing up clinical cases/manuscripts | No | | 159 (89%) | |
|  | Yes | | 20 (11%) | |
| Writing up clinical study IRB applications | No | | 169 (94%) | |
|  | Yes | | 10 (6%) | |
| Following up patient care needs online/virtually | No | | 166 (93%) | |
|  | Yes | | 13 (7%) | |
| Volunteer work (assist at nursing homes, shelters, non patient care, hospital processes, etc) | No | | 126 (70%) | |
|  | Yes | | 53 (30%) | |
| Help out with COVID-19-related research | No | | 157 (88%) | |
|  | Yes | | 22 (12%) | |
| Help with patient care | No | | 131 (73%) | |
|  | Yes | | 48 (27%) | |
| Virtual patient encounters are as good as in-person patient encounters for my medical training | not SA/A | | 168 (94%) | |
|  | Strongly agree/ Agree | | 11 (6%) | |
| My research productivity/medical training will be negatively impacted in the short-term | not SA/A | | 67 (37%) | |
|  | Strongly agree/ Agree | | 112 (63%) | |
| My research productivity/medical training will be negatively impacted in the long-term | not SA/A | | 129 (72%) | |
|  | Strongly agree/ Agree | | 50 (28%) | |
| My medical training has not been compromised and I will be on track for graduating | not SA/A | | 80 (45%) | |
|  | Strongly agree/ Agree | | 99 (55%) | |
| I have changed my research efforts to focus on COVID-19 related topics | not SA/A | | 166 (93%) | |
|  | Strongly agree/ Agree | | 13 (7%) | |
| I have changed my intended career path/specialty intentions as a result of COVID-19 | not SA/A | | 166 (93%) | |
|  | Strongly agree/ Agree | | 13 (7%) | |
| I am concerned about my medical training being compromised and not being prepared for internship/residency | not SA/A | | 106 (59%) | |
|  | Strongly agree/ Agree | | 73 (41%) | |
| The COVID-19 pandemic has caused me a significant amount of stress, anxiety, hopelessness and/or depression | not SA/A | | 87 (49%) | |
|  | Strongly agree/ Agree | | 92 (51%) | |
| The COVID-19 pandemic has caused sleep problems, decreased energy, changes in appetite, difficulty concentrating and/or restlessness | not SA/A | | 90 (50%) | |
|  | Strongly agree/ Agree | | 89 (50%) | |
| Uncertainty of not being able to finish my research or to graduate is a great source of stress | not SA/A | | 137 (77%) | |
|  | Strongly agree/ Agree | | 42 (23%) | |
| Worrying about my own health is a great source of stress | not SA/A | | 123 (69%) | |
|  | Strongly agree/ Agree | | 56 (31%) | |
| Worrying about the health of my family/friends is a great source of stress | not SA/A | | 66 (37%) | |
|  | Strongly agree/ Agree | | 113 (63%) | |
| I am worried about my own health from direct patient contact of confirmed or suspected COVID-19 patients | not SA/A | | 136 (76%) | |
|  | Strongly agree/ Agree | | 43 (24%) | |
| I am worried about my own health from casual contact in the public | not SA/A | | 108 (60%) | |
|  | Strongly agree/ Agree | | 71 (40%) | |
| Social isolation is a source of a great deal of stress | not SA/A | | 69 (39%) | |
|  | Strongly agree/ Agree | | 110 (61%) | |
| Financial consequences of the pandemic cause a great deal of stress | not SA/A | | 140 (78%) | |
|  | Strongly agree/ Agree | | 39 (22%) | |
| I am stressed out due to the political climate around health disparities and gender inequalities | not SA/A | | 56 (31%) | |
|  | Strongly agree/ Agree | | 123 (69%) | |
| I am stressed out due to how the pandemic has been managed at the governmental level | not SA/A | | 48 (27%) | |
|  | Strongly agree/ Agree | | 131 (73%) | |
| I am stressed out due to how the pandemic has been managed at the local level | not SA/A | | 81 (45%) | |
|  | Strongly agree/ Agree | | 98 (55%) | |
| I have been implementing stress-relieving practices and I feel that I have a handle on my stress levels | not SA/A | | 80 (45%) | |
|  | Strongly agree/ Agree | | 99 (55%) | |
| I am worried about the long-term effects that COVID-19 will have on my career, personal life, and/or family/friends | not SA/A | | 90 (50%) | |
|  | Strongly agree/ Agree | | 89 (50%) | |
| I am optimistic about the future given the trajectory of the COVID-19 pandemic | not SA/A | | 113 (63%) | |
|  | Strongly agree/ Agree | | 66 (37%) | |
| stress outcome | Neither Agree nor Disagree | | 1 (1%) | |
|  | No | | 26 (15%) | |
|  | Yes | | 152 (85%) | |
| policy related stress | Neither Agree nor Disagree | | 11 (6%) | |
|  | No | | 20 (11%) | |
|  | Yes | | 148 (83%) | |
| productivity outcome | Neither Agree nor Disagree | | 23 (13%) | |
|  | No | | 39 (22%) | |
|  | Yes | | 117 (65%) | |
| optimism outcome | Neither Agree nor Disagree | | 63 (35%) | |
|  | No | | 50 (28%) | |
|  | Yes | | 66 (37%) | |
|  |  | |  | |
| **Graduate students** | |  | |  |
|  | Total: **(n = 320)** | |  | |
| Age | Mean (SD) | | 27 61 (2 57) | |
|  | Median (IQR) | | 27 (26, 29) | |
|  | Range | | (21, 42) | |
| Gender | Female | | 158 (49%) | |
|  | Male/Other | | 162 (51%) | |
| Do you identify as transgender | No | | 311 (97%) | |
|  | Yes | | 5 (2%) | |
| American Indian or Alaskan Native | No | | 313 (98%) | |
|  | Yes | | 7 (2%) | |
| Black or African American | No | | 307 (96%) | |
|  | Yes | | 13 (4%) | |
| Chinese | No | | 289 (90%) | |
|  | Yes | | 31 (10%) | |
| Vietnamese | No | | 318 (99%) | |
|  | Yes | | 2 (1%) | |
| Korean | No | | 310 (97%) | |
|  | Yes | | 10 (3%) | |
| Japanese | No | | 317 (99%) | |
|  | Yes | | 3 (1%) | |
| Asian Indian | No | | 296 (92%) | |
|  | Yes | | 24 (8%) | |
| Filipino | No | | 314 (98%) | |
|  | Yes | | 6 (2%) | |
| Other Asian | No | | 307 (96%) | |
|  | Yes | | 13 (4%) | |
| Native Hawaiian or Other Pacific Islander | No | | 320 (100%) | |
| White | No | | 123 (38%) | |
|  | Yes | | 197 (62%) | |
| Multi-racial | No | | 297 (93%) | |
|  | Yes | | 23 (7%) | |
| Ethnicity | Hispanic or Latino/a | | 19 (6%) | |
|  | Not/other | | 300 (94%) | |
| Are you married or partnered | No | | 182 (57%) | |
|  | Other (please specify) | | 3 (1%) | |
|  | Yes | | 135 (42%) | |
| Do you have children | No | | 290 (91%) | |
|  | Other (please specify) | | 3 (1%) | |
|  | Yes | | 27 (8%) | |
| Region | Midwest | | 90 (28%) | |
|  | Northeast | | 113 (35%) | |
|  | Northwest/Southwest | | 35 (11%) | |
|  | South/Southeast | | 82 (26%) | |
| Is your institution public or private | Private | | 160 (50%) | |
|  | Public | | 160 (50%) | |
| Dual degree | No | | 14 (4%) | |
|  | Yes | | 306 (96%) | |
| Have you received a COVID 19 vaccination | No | | 3 (1%) | |
|  | Yes | | 317 (99%) | |
|  | Yes | | 2 (1%) | |
| Training year | Graduate School-Year 1 | | 77 (24%) | |
|  | Graduate School-Year 2 | | 65 (20%) | |
|  | Graduate School-Year 3 | | 73 (23%) | |
|  | Graduate School-Year 4 | | 69 (22%) | |
|  | Graduate School-Year 5 | | 25 (8%) | |
|  | Graduate School-Year 6+ | | 9 (3%) | |
|  | Not applicable | | 2 (1%) | |
| Type of lab Dry lab | No | | 229 (72%) | |
|  | Yes | | 91 (28%) | |
| Type of lab Wet lab | No | | 55 (17%) | |
|  | Yes | | 265 (83%) | |
| Type of lab Social Sciences | No | | 306 (96%) | |
|  | Yes | | 14 (4%) | |
| Field of research Biological Sciences (Environmental biology, Molecular Cellular Biosciences, Bioengineering) | No | | 40 (12%) | |
|  | Yes | | 280 (88%) | |
| Field of research Computer and Information Science and Engineering (Computer and Network Systems, Information and Intelligent Systems) | No | | 293 (92%) | |
|  | Yes | | 27 (8%) | |
| Field of research Education and Human Resources (Undergraduate, Graduate Education, Medical Education, Human Resource Development) | No | | 318 (99%) | |
|  | Yes | | 2 (1%) | |
| Field of research Geosciences (Atmospheric, Geospace Sciences, Earth Sciences, Ocean Sciences) | No | | 319 (100%) | |
|  | Yes | | 1 (0%) | |
| Field of research Mathematical and Physical Sciences (Chemistry, Statistics, Biostatistics, Epidemiology, Materials Research, Physics, Astronomical Sciences) | No | | 296 (92%) | |
|  | Yes | | 24 (8%) | |
| Field of research Social, Behavioral, and Economic Sciences (Behavioral and Cognitive Sciences, Social and Economic Sciences ) | No | | 300 (94%) | |
|  | Yes | | 20 (6%) | |
| My lab has shut down | No | | 214 (67%) | |
|  | Yes | | 106 (33%) | |
| Experiments have been delayed or impaired | No | | 62 (19%) | |
|  | Yes | | 258 (81%) | |
| I am unable to perform any research-related activities | No | | 283 (88%) | |
|  | Yes | | 37 (12%) | |
| My qualifying exam or thesis defense has been postponed | No | | 279 (87%) | |
|  | Yes | | 41 (13%) | |
| My qualifying exam or thesis defense has been converted into a virtual platform (ie Zoom, Gotomeeting) | No | | 172 (54%) | |
|  | Yes | | 148 (46%) | |
| My transition back to medical school training has been delayed or impaired | No | | 275 (86%) | |
|  | Yes | | 45 (14%) | |
| My visa is threatened due to an inability to conduct research | No | | 320 (100%) | |
| Graduate training affected by campus libraries and/or computer centers closures | No | | 207 (65%) | |
|  | Yes | | 113 (35%) | |
| Proportion of time spending on Childcare/homeschooling | >0 | | 29 (12%) | |
|  | 0 | | 223 (88%) | |
| Proportion of time spending on Clinical duties | <=45% | | 263 (82%) | |
|  | >45% | | 57 (18%) | |
| Proportion of time spending on Administrative duties | <=45% | | 270 (84%) | |
|  | >45% | | 50 (16%) | |
| Proportion of time spending on Research/scholarly activities | <=45% | | 58 (18%) | |
|  | >45% | | 262 (82%) | |
| Proportion of time spending on Personal time | <=45% | | 306 (96%) | |
|  | >45% | | 14 (4%) | |
| Proportion of time spending on Volunteer activities | <=45% | | 296 (92%) | |
|  | >45% | | 24 (8%) | |
| Personal life affected by campus transportation or local transit being shut down | No | | 265 (83%) | |
|  | Yes | | 55 (17%) | |
| Personal life affected by childcare facilities being shut down | No | | 308 (96%) | |
|  | Yes | | 12 (4%) | |
| Personal life affected by personally taking care of my children since daycare facilities have closed/have no alternate source of childcare | No | | 309 (97%) | |
|  | Yes | | 11 (3%) | |
| I am homeschooling my children | No | | 316 (99%) | |
|  | Yes | | 4 (1%) | |
| I am taking care of elderly loved ones | No | | 319 (100%) | |
|  | Yes | | 1 (0%) | |
| Personal life affected by campus libraries closures | No | | 223 (70%) | |
|  | Yes | | 97 (30%) | |
| Personal life affected by campus computer center closures | No | | 265 (83%) | |
|  | Yes | | 55 (17%) | |
| I am working from home | No | | 134 (42%) | |
|  | Yes | | 186 (58%) | |
| I have lost my job due to the pandemic | No | | 318 (99%) | |
|  | Yes | | 2 (1%) | |
| I have been physically isolated from friends/family due to my work | No | | 137 (43%) | |
|  | Yes | | 183 (57%) | |
| I have still been able to keep in touch with friends/family via virtual platforms | No | | 68 (21%) | |
|  | Yes | | 252 (79%) | |
| I am living by myself thus am not concerned about quarantining from family members/roommates/partner | No | | 261 (82%) | |
|  | Yes | | 59 (18%) | |
| I have to self-quarantine away from my family/roommates/partner due to being exposed to COVID-19 or because of COVID-19-related symptoms (without confirmatory COVID-19 testing) | No | | 285 (89%) | |
|  | Yes | | 35 (11%) | |
| I have been exposed to COVID-19 or have symptoms (without confirmatory COVID-19 testing), but have no option to live in a different residence (hotel, institution facility) | No | | 294 (92%) | |
|  | Yes | | 26 (8%) | |
| I have been exposed to COVID-19 or have symptoms (without confirmatory COVID-19 testing), and have been offered an alternative residence (hotel, institution facility) | No | | 320 (100%) | |
| I have to self-quarantine away from my family/roommates/partner due to being exposed to COVID-19 and have tested positive for COVID-19 | No | | 313 (98%) | |
|  | Yes | | 7 (2%) | |
| I am currently being treated for symptoms/sequelae of being infected with COVID-19 | No | | 319 (100%) | |
|  | Yes | | 1 (0%) | |
| I have accrued significant medical debt after being treated for symptoms/sequelae of being infected with COVID-19 | No | | 317 (99%) | |
|  | Yes | | 3 (1%) | |
| My family/roommates/partner have been affected or infected because of my COVID-19 infection | No | | 300 (94%) | |
|  | Yes | | 20 (6%) | |
| Personal life has not been affected by COVID-19 | No | | 300 (94%) | |
|  | Yes | | 20 (6%) | |
| Top 3 increase (onset) career success | No | | 240 (75%) | |
|  | Yes | | 80 (25%) | |
| Top 3 increase (onset) research | No | | 243 (76%) | |
|  | Yes | | 77 (24%) | |
| Top 3 increase (onset) friends/family | No | | 106 (33%) | |
|  | Yes | | 214 (67%) | |
| Top 3 increase (onset) time w/my children | No | | 298 (93%) | |
|  | Yes | | 22 (7%) | |
| Top 3 increase (onset) time w/my partner | No | | 166 (52%) | |
|  | Yes | | 154 (48%) | |
| Top 3 increase (onset) personal health | No | | 123 (38%) | |
|  | Yes | | 197 (62%) | |
| Top 3 increase (onset) exercise | No | | 192 (60%) | |
|  | Yes | | 128 (40%) | |
| Top 3 increase (onset) spending more time with hobbies | No | | 204 (64%) | |
|  | Yes | | 116 (36%) | |
| Working on data analysis and design of experiments | No | | 35 (11%) | |
|  | Yes | | 285 (89%) | |
| Reading scientific literature | No | | 47 (15%) | |
|  | Yes | | 273 (85%) | |
| Attending journal clubs by virtual platform | No | | 131 (41%) | |
|  | Yes | | 189 (59%) | |
| Attending lab meetings by virtual platform | No | | 81 (25%) | |
|  | Yes | | 239 (75%) | |
| Preparing drafts of manuscripts | No | | 132 (41%) | |
|  | Yes | | 188 (59%) | |
| Preparing grant/fellowship/faculty applications | No | | 169 (53%) | |
|  | Yes | | 151 (47%) | |
| Preparing dissertation chapters | No | | 263 (82%) | |
|  | Yes | | 57 (18%) | |
| Preparing review articles | No | | 211 (66%) | |
|  | Yes | | 109 (34%) | |
| Preparing research seminars and/or posters for meetings/conferences | No | | 202 (63%) | |
|  | Yes | | 118 (37%) | |
| Taking online courses to enhance skills | No | | 229 (72%) | |
|  | Yes | | 91 (28%) | |
| Performing computational modeling | No | | 263 (82%) | |
|  | Yes | | 57 (18%) | |
| Search/analysis of genomic/proteomic sequence data | No | | 250 (78%) | |
|  | Yes | | 70 (22%) | |
| Secondary data analysis | No | | 234 (73%) | |
|  | Yes | | 86 (27%) | |
| Working collaboratively to outline an experimental plan for a study | No | | 250 (78%) | |
|  | Yes | | 70 (22%) | |
| Preparing figures or text for a collaborative manuscript | No | | 202 (63%) | |
|  | Yes | | 118 (37%) | |
| Enhancing career development through institutional/online resources | No | | 272 (85%) | |
|  | Yes | | 48 (15%) | |
| Taking practice questions, reviewing board material for exams | No | | 263 (82%) | |
|  | Yes | | 57 (18%) | |
| Volunteer work (assist at nursing homes, helping at shelters, non patient care, hospital processes, etc) | No | | 239 (75%) | |
|  | Yes | | 81 (25%) | |
| Help with COVID-19 related research | No | | 273 (85%) | |
|  | Yes | | 47 (15%) | |
| Help with patient care | No | | 289 (90%) | |
|  | Yes | | 31 (10%) | |
| Virtual classrooms recapitulate in-person learning | not SA/A | | 270 (84%) | |
|  | Strongly agree/ Agree | | 50 (16%) | |
| Virtual patient encounters are as good as in-person patient encounters for my medical training | not SA/A | | 312 (98%) | |
|  | Strongly agree/ Agree | | 8 (2%) | |
| My research productivity/medical training will be negatively impacted in the short-term | not SA/A | | 84 (26%) | |
|  | Strongly agree/ Agree | | 236 (74%) | |
| My research productivity/medical training will be negatively impacted in the long-term | not SA/A | | 161 (50%) | |
|  | Strongly agree/ Agree | | 159 (50%) | |
| I have changed my research efforts to focus on COVID-19 related topics | not SA/A | | 289 (90%) | |
|  | Strongly agree/ Agree | | 31 (10%) | |
| I have changed my intended career path/specialty intentions as a result of COVID-19 | not SA/A | | 294 (92%) | |
|  | Strongly agree/ Agree | | 26 (8%) | |
| The COVID-19 pandemic has caused me a significant amount of stress, anxiety, hopelessness and/or depression | not SA/A | | 119 (37%) | |
|  | Strongly agree/ Agree | | 201 (63%) | |
| The COVID-19 pandemic has caused sleep problems, decreased energy, changes in appetite, difficulty concentrating and/or restlessness | not SA/A | | 129 (40%) | |
|  | Strongly agree/ Agree | | 191 (60%) | |
| Uncertainty of not being able to finish my research or to graduate is a great source of stress | not SA/A | | 146 (46%) | |
|  | Strongly agree/ Agree | | 174 (54%) | |
| Worrying about my own health is a great source of stress | not SA/A | | 195 (61%) | |
|  | Strongly agree/ Agree | | 125 (39%) | |
| Worrying about the health of my family/friends is a great source of stress | not SA/A | | 93 (29%) | |
|  | Strongly agree/ Agree | | 227 (71%) | |
| I am worried about my own health from direct patient contact of confirmed or suspected COVID-19 patients | not SA/A | | 286 (89%) | |
|  | Strongly agree/ Agree | | 34 (11%) | |
| I am worried about my own health from casual contact in the public | not SA/A | | 180 (56%) | |
|  | Strongly agree/ Agree | | 140 (44%) | |
| Social isolation is a source of a great deal of stress | not SA/A | | 127 (40%) | |
|  | Strongly agree/ Agree | | 193 (60%) | |
| Financial consequences of the pandemic is a source of a great deal of stress | not SA/A | | 229 (72%) | |
|  | Strongly agree/ Agree | | 91 (28%) | |
| I am stressed out due to the political climate around health disparities and gender inequalities | not SA/A | | 124 (39%) | |
|  | Strongly agree/ Agree | | 196 (61%) | |
| I am stressed out due to how the pandemic has been managed at the governmental level | not SA/A | | 80 (25%) | |
|  | Strongly agree/ Agree | | 240 (75%) | |
| I am stressed out due to how the pandemic has been managed at the local level | not SA/A | | 138 (43%) | |
|  | Strongly agree/ Agree | | 182 (57%) | |
| I have been implementing stress-relieving practices, and I feel that I have a handle on my stress levels | not SA/A | | 170 (53%) | |
|  | Strongly agree/ Agree | | 150 (47%) | |
| I am worried about the long-term effects that COVID-19 will have on my career, personal life, and/or family/friends | not SA/A | | 148 (46%) | |
|  | Strongly agree/ Agree | | 172 (54%) | |
| I am optimistic about the future given the trajectory of the COVID-19 pandemic | not SA/A | | 199 (62%) | |
|  | Strongly agree/ Agree | | 121 (38%) | |
| stress outcome | No | | 42 (13%) | |
|  | Yes | | 277 (87%) | |
| policy related stress | Neither Agree nor Disagree | | 10 (3%) | |
|  | No | | 52 (16%) | |
|  | Yes | | 257 (81%) | |
| productivity outcome | Neither Agree nor Disagree | | 21 (7%) | |
|  | No | | 46 (14%) | |
|  | Yes | | 251 (79%) | |
| optimism outcome | Neither Agree nor Disagree | | 95 (30%) | |
|  | No | | 99 (31%) | |
|  | Yes | | 121 (38%) | |
|  |  | |  | |
| **Residents/fellows/faculty** | |  | |  |
|  | **Total: n=178** | |  | |
| Age | Mean (SD) | | 35 65 (5 7) | |
|  | Median (IQR) | | 35 (32, 39) | |
|  | Range | | (19, 51) | |
| Gender | Female | | 90 (51%) | |
|  | Male/Other | | 88 (49%) | |
| Do you identify as transgender | No | | 178 (100%) | |
| American Indian or Alaskan Native | No | | 177 (99%) | |
|  | Yes | | 1 (1%) | |
| Black or African American | No | | 173 (97%) | |
|  | Yes | | 5 (3%) | |
| Chinese | No | | 161 (90%) | |
|  | Yes | | 17 (10%) | |
| Vietnamese | No | | 178 (100%) | |
| Korean | No | | 171 (96%) | |
|  | Yes | | 7 (4%) | |
| Japanese | No | | 177 (99%) | |
|  | Yes | | 1 (1%) | |
| Asian Indian | No | | 169 (95%) | |
|  | Yes | | 9 (5%) | |
| Filipino | No | | 178 (100%) | |
| Other Asian | No | | 174 (98%) | |
|  | Yes | | 4 (2%) | |
| Native Hawaiian or Other Pacific Islander | No | | 177 (99%) | |
|  | Yes | | 1 (1%) | |
| White | No | | 65 (37%) | |
|  | Yes | | 113 (63%) | |
| Multi-racial | No | | 169 (95%) | |
|  | Yes | | 9 (5%) | |
| Prefer Not to Answer | No | | 168 (94%) | |
|  | Yes | | 10 (6%) | |
| Other (please specify) | No | | 173 (97%) | |
|  | Yes | | 5 (3%) | |
| ethnicity | Hispanic or Latino/a | | 16 (9%) | |
|  | Not/other | | 162 (91%) | |
| Are you married or partnered | No | | 46 (26%) | |
|  | Yes | | 131 (74%) | |
| Do you have children | No | | 93 (52%) | |
|  | Yes | | 85 (48%) | |
| Region | Midwest | | 60 (34%) | |
|  | Northeast | | 59 (33%) | |
|  | Northwest/Southwest | | 23 (13%) | |
|  | South/Southeast | | 34 (19%) | |
| Is your institution public or private | Private | | 113 (63%) | |
|  | Public | | 65 (37%) | |
| Dual degree | No | | 72 (40%) | |
|  | Yes | | 106 (60%) | |
| Have you received a COVID 19 vaccination | No | | 2 (1%) | |
|  | Yes | | 176 (99%) | |
| Training year | Faculty | | 65 (37%) | |
|  | Fellowship | | 42 (24%) | |
|  | Internship | | 12 (7%) | |
|  | Not applicable | | 1 (1%) | |
|  | Other (please specify) | | 1 (1%) | |
|  | Post-doctoral research | | 11 (6%) | |
|  | Post-graduate Non-academic | | 3 (2%) | |
|  | Residency | | 43 (24%) | |
| Specialty | Anesthesiology | | 12 (7%) | |
|  | Colon and rectal surgery | | 1 (1%) | |
|  | Dermatology | | 8 (4%) | |
|  | Emergency medicine | | 1 (1%) | |
|  | Family Medicine | | 1 (1%) | |
|  | General surgery | | 2 (1%) | |
|  | IM: Allergy/immunology | | 11 (6%) | |
|  | IM: Cardiology | | 9 (5%) | |
|  | IM: Endocrinology | | 4 (2%) | |
|  | IM: Gastroenterology | | 2 (1%) | |
|  | IM: Geriatrics | | 1 (1%) | |
|  | IM: Hematology, Oncology | | 18 (10%) | |
|  | IM: Infectious Disease | | 6 (3%) | |
|  | IM: Nephrology | | 1 (1%) | |
|  | IM: Pulmonology/Critical care | | 5 (3%) | |
|  | IM: Rheumatology | | 7 (4%) | |
|  | Internal Medicine | | 24 (13%) | |
|  | Medical Genetics | | 2 (1%) | |
|  | Neurological surgery | | 1 (1%) | |
|  | Neurology | | 9 (5%) | |
|  | Ob/Gynecology | | 3 (2%) | |
|  | Orthopedic surgery | | 4 (2%) | |
|  | Other (please specify) | | 6 (3%) | |
|  | Otolaryngology | | 5 (3%) | |
|  | Pathology | | 7 (4%) | |
|  | Pediatrics | | 19 (11%) | |
|  | Plastic surgery | | 1 (1%) | |
|  | Psychiatry | | 5 (3%) | |
|  | Radiation oncology | | 2 (1%) | |
|  | Radiology | | 1 (1%) | |
| Are you in a tenure track position | No | | 110 (73%) | |
|  | Yes | | 35 (23%) | |
| If you are in a tenure track position, are you worried about your tenure clock due to this pandemic | My institution has reset the tenure clock due to the pandemic | | 18 (10%) | |
|  | No | | 50 (28%) | |
|  | Yes | | 98 (55%) | |
|  | Yes | | 12 (7%) | |
| Type of laboratory Dry lab | No | | 128 (72%) | |
|  | Yes | | 50 (28%) | |
| Type of laboratory Wet lab | No | | 78 (44%) | |
|  | Yes | | 100 (56%) | |
| Type of laboratory Social Sciences | No | | 160 (90%) | |
|  | Yes | | 18 (10%) | |
| Field of research Biological Sciences (Environmental biology, Molecular Cellular Biosciences, Bioengineering) | No | | 54 (30%) | |
|  | Yes | | 124 (70%) | |
| Field of research Computer and Information Science and Engineering (Computer and Network Systems, Information and Intelligent Systems) | No | | 169 (95%) | |
|  | Yes | | 9 (5%) | |
| Field of research Education and Human Resources (Undergraduate, Graduate Education, Medical Education, Human Resource Development) | No | | 167 (94%) | |
|  | Yes | | 11 (6%) | |
| Field of research Geosciences (Atmospheric, Geospace Sciences, Earth Sciences, Ocean Sciences) | No | | 178 (100%) | |
| Field of research Mathematical and Physical Sciences (Chemistry, Statistics, Biostatistics, Epidemiology, Materials Research, Physics, Astronomical Sciences) | No | | 167 (94%) | |
|  | Yes | | 11 (6%) | |
| Field of research Social, Behavioral, and Economic Sciences (Behavioral and Cognitive Sciences, Social and Economic Sciences ) | No | | 162 (91%) | |
|  | Yes | | 16 (9%) | |
| My institution has offered me hazard pay for my frontline efforts in the COVID 19 pandemic | No | | 151 (85%) | |
|  | Yes | | 15 (8%) | |
| Proportion of time spending on Childcare/homeschooling | >0 | | 84 (57%) | |
|  | 0 | | 64 (43%) | |
| Proportion of time spending on Clinical duties | <=45% | | 89 (50%) | |
|  | >45% | | 89 (50%) | |
| Proportion of time spending on Administrative duties | <=45% | | 151 (85%) | |
|  | >45% | | 27 (15%) | |
| Proportion of time spending on Research/scholarly activities | <=45% | | 113 (63%) | |
|  | >45% | | 65 (37%) | |
| Proportion of time spending on Personal time | <=45% | | 166 (93%) | |
|  | >45% | | 12 (7%) | |
| Proportion of time spending on Volunteer activities | <=45% | | 159 (89%) | |
|  | >45% | | 19 (11%) | |
| I am no longer seeing patients in person | No | | 170 (96%) | |
|  | Yes | | 8 (4%) | |
| I am seeing inpatients in a virtual manner | No | | 165 (93%) | |
|  | Yes | | 13 (7%) | |
| I am seeing outpatients in a virtual manner | No | | 101 (57%) | |
|  | Yes | | 77 (43%) | |
| I have been redeployed to care for inpatients | No | | 165 (93%) | |
|  | Yes | | 13 (7%) | |
| I am providing care to my patients in a different clinical discipline than my own or have been pulled to care for COVID-19 patients | No | | 162 (91%) | |
|  | Yes | | 16 (9%) | |
| Elective procedures/surgeries have been canceled | No | | 131 (74%) | |
|  | Yes | | 47 (26%) | |
| My research lab has shut down | No | | 142 (80%) | |
|  | Yes | | 36 (20%) | |
| My lab personnel are no longer able to perform research | No | | 152 (85%) | |
|  | Yes | | 26 (15%) | |
| Personal life affected by campus transportation or local transit being shut down | No | | 162 (91%) | |
|  | Yes | | 16 (9%) | |
| Personal life affected by childcare facilities being shut down | No | | 139 (78%) | |
|  | Yes | | 39 (22%) | |
| I have been personally taking care of my children since daycare facilities have closed/have no alternate source of childcare | No | | 150 (84%) | |
|  | Yes | | 28 (16%) | |
| I am homeschooling my children | No | | 159 (89%) | |
|  | Yes | | 19 (11%) | |
| I am taking care of elderly loved ones | No | | 178 (100%) | |
| Personal life affected by campus libraries being closed | No | | 132 (74%) | |
|  | Yes | | 46 (26%) | |
| Personal life affected by campus computer centers being closed | No | | 163 (92%) | |
|  | Yes | | 15 (8%) | |
| I am working from home | No | | 109 (61%) | |
|  | Yes | | 69 (39%) | |
| I have lost my job due to the pandemic | No | | 177 (99%) | |
|  | Yes | | 1 (1%) | |
| I have gotten a pay/salary cut | No | | 160 (90%) | |
|  | Yes | | 18 (10%) | |
| My pay/salary has maintained the same during this pandemic | No | | 93 (52%) | |
|  | Yes | | 85 (48%) | |
| I have been physically isolated from friends/family due to my work | No | | 82 (46%) | |
|  | Yes | | 96 (54%) | |
| I have still been able to keep in touch with friends/family via virtual platforms | No | | 63 (35%) | |
|  | Yes | | 115 (65%) | |
| I am living by myself thus am not concerned about quarantining from family members/roommates/partner | No | | 152 (85%) | |
|  | Yes | | 26 (15%) | |
| I have to self-quarantine away from my family/roommates/partner due to being exposed to COVID-19 or because of COVID-19-related symptoms (without confirmatory COVID-19 testing) | No | | 155 (87%) | |
|  | Yes | | 23 (13%) | |
| I have been exposed to COVID-19 or have symptoms (without confirmatory COVID-19 testing), but have no option to live in a different residence (hotel, institution facility) | No | | 156 (88%) | |
|  | Yes | | 22 (12%) | |
| I have been exposed to COVID-19 or have symptoms (without confirmatory COVID-19 testing), and have been offered an alternative residence (hotel, institution facility) | No | | 173 (97%) | |
|  | Yes | | 5 (3%) | |
| I have to self-quarantine away from my family/roommates/partner due to being exposed to COVID-19 and have tested positive for COVID-19 | No | | 169 (95%) | |
|  | Yes | | 9 (5%) | |
| I am currently being treated for symptoms/sequelae of being infected with COVID-19 | No | | 177 (99%) | |
|  | Yes | | 1 (1%) | |
| I have accrued significant medical debt after being treated for symptoms/sequelae of being infected with COVID-19 | No | | 178 (100%) | |
| My family/roommates/partner have been affected or infected because of my COVID-19 infection | No | | 165 (93%) | |
|  | Yes | | 13 (7%) | |
| My personal life has not been affected by the pandemic | No | | 175 (98%) | |
|  | Yes | | 3 (2%) | |
| Top 3 increase (onset) career success | No | | 143 (80%) | |
|  | Yes | | 35 (20%) | |
| Top 3 increase (onset) research | No | | 144 (81%) | |
|  | Yes | | 34 (19%) | |
| Top 3 increase (onset) friends/family | No | | 62 (35%) | |
|  | Yes | | 116 (65%) | |
| Top 3 increase (onset) time w/my children | No | | 112 (63%) | |
|  | Yes | | 66 (37%) | |
| Top 3 increase (onset) time w/my partner | No | | 94 (53%) | |
|  | Yes | | 84 (47%) | |
| Top 3 increase (onset) personal health | No | | 90 (51%) | |
|  | Yes | | 88 (49%) | |
| Top 3 increase (onset) exercise | No | | 124 (70%) | |
|  | Yes | | 54 (30%) | |
| Top 3 increase (onset) spending more time with hobbies | No | | 145 (81%) | |
|  | Yes | | 33 (19%) | |
| Working on data analysis and design of experiments | No | | 84 (47%) | |
|  | Yes | | 94 (53%) | |
| Reading scientific literature | No | | 58 (33%) | |
|  | Yes | | 120 (67%) | |
| Attending journal clubs by virtual platform | No | | 97 (54%) | |
|  | Yes | | 81 (46%) | |
| Attending lab meetings by virtual platform | No | | 93 (52%) | |
|  | Yes | | 85 (48%) | |
| Preparing drafts of manuscripts | No | | 72 (40%) | |
|  | Yes | | 106 (60%) | |
| Preparing grant/fellowship/faculty applications | No | | 97 (54%) | |
|  | Yes | | 81 (46%) | |
| Preparing dissertation chapters | No | | 178 (100%) | |
| Preparing review articles | No | | 122 (69%) | |
|  | Yes | | 56 (31%) | |
| Preparing research seminars and/or posters for meetings/conferences | No | | 131 (74%) | |
|  | Yes | | 47 (26%) | |
| Taking online courses to enhance skills | No | | 139 (78%) | |
|  | Yes | | 39 (22%) | |
| Perform computational modeling | No | | 161 (90%) | |
|  | Yes | | 17 (10%) | |
| Search/analysis of genomic/proteomic sequence data | No | | 161 (90%) | |
|  | Yes | | 17 (10%) | |
| Secondary data analysis | No | | 139 (78%) | |
|  | Yes | | 39 (22%) | |
| Working collaboratively to outline an experimental plan for a study | No | | 138 (78%) | |
|  | Yes | | 40 (22%) | |
| Preparing figures or text for a collaborative manuscript | No | | 125 (70%) | |
|  | Yes | | 53 (30%) | |
| Enhancing career development through institutional/online resources | No | | 146 (82%) | |
|  | Yes | | 32 (18%) | |
| Taking practice questions, reviewing board material for my specialty exam(s) | No | | 119 (67%) | |
|  | Yes | | 59 (33%) | |
| Writing up clinical cases/manuscripts | No | | 134 (75%) | |
|  | Yes | | 44 (25%) | |
| Writing up clinical study IRB applications | No | | 135 (76%) | |
|  | Yes | | 43 (24%) | |
| Following up patient care needs online/virtually | No | | 120 (67%) | |
|  | Yes | | 58 (33%) | |
| Volunteer work (assist at nursing homes, helping at shelters, non patient care, hospital processes, etc) | No | | 168 (94%) | |
|  | Yes | | 10 (6%) | |
| Help with COVID-19-related research | No | | 142 (80%) | |
|  | Yes | | 36 (20%) | |
| Help with patient care | No | | 91 (51%) | |
|  | Yes | | 87 (49%) | |
| Virtual patient encounters are as good as in-person patient encounters for my medical training | not SA/A | | 156 (88%) | |
|  | Strongly agree/ Agree | | 22 (12%) | |
| Virtual patient encounters are as good as in-person patient encounters for patient care | not SA/A | | 158 (89%) | |
|  | Strongly agree/ Agree | | 20 (11%) | |
| My research productivity/medical training will be negatively impacted in the short-term | not SA/A | | 53 (30%) | |
|  | Strongly agree/ Agree | | 125 (70%) | |
| My research productivity/medical training will be negatively impacted in the long-term | not SA/A | | 88 (49%) | |
|  | Strongly agree/ Agree | | 90 (51%) | |
| I have changed my research efforts to focus on COVID-19 related topics | not SA/A | | 134 (75%) | |
|  | Strongly agree/ Agree | | 44 (25%) | |
| I feel like my patients will suffer due to delayed presentation and/or disrupted in-person follow up | not SA/A | | 59 (33%) | |
|  | Strongly agree/ Agree | | 119 (67%) | |
| I am concerned about not being able to find a full-time job due to university/health system hiring freezes | not SA/A | | 137 (77%) | |
|  | Strongly agree/ Agree | | 41 (23%) | |
| The COVID-19 pandemic has caused me a significant amount of stress, anxiety, hopelessness and/or depression | not SA/A | | 77 (43%) | |
|  | Strongly agree/ Agree | | 101 (57%) | |
| The COVID-19 pandemic has caused sleep problems, decreased energy, changes in appetite, difficulty concentrating and/or restlessness | not SA/A | | 100 (56%) | |
|  | Strongly agree/ Agree | | 78 (44%) | |
| Uncertainty of not being able to finish my research or to graduate is a great source of stress | not SA/A | | 102 (57%) | |
|  | Strongly agree/ Agree | | 76 (43%) | |
| Worrying about my own health is a great source of stress | not SA/A | | 110 (62%) | |
|  | Strongly agree/ Agree | | 68 (38%) | |
| Worrying about the health of my family/friends is a great source of stress | not SA/A | | 54 (30%) | |
|  | Strongly agree/ Agree | | 124 (70%) | |
| I am worried about my own health from direct patient contact of confirmed or suspected COVID-19 patients | not SA/A | | 106 (60%) | |
|  | Strongly agree/ Agree | | 72 (40%) | |
| I am worried about my own health from casual contact in the public | not SA/A | | 85 (48%) | |
|  | Strongly agree/ Agree | | 93 (52%) | |
| Social isolation is a source of a great deal of stress | not SA/A | | 79 (44%) | |
|  | Strongly agree/ Agree | | 99 (56%) | |
| Financial consequences of the pandemic is a source of a great deal of stress | not SA/A | | 132 (74%) | |
|  | Strongly agree/ Agree | | 46 (26%) | |
| I am stressed out due to the political climate around health disparities and gender inequalities | not SA/A | | 71 (40%) | |
|  | Strongly agree/ Agree | | 107 (60%) | |
| I am stressed out due to how the pandemic has been managed at the governmental level | not SA/A | | 43 (24%) | |
|  | Strongly agree/ Agree | | 135 (76%) | |
| I am stressed out due to how the pandemic has been managed at the local level | not SA/A | | 77 (43%) | |
|  | Strongly agree/ Agree | | 101 (57%) | |
| I have been implementing stress-relieving practices and I feel that I have a handle on my stress levels | not SA/A | | 102 (57%) | |
|  | Strongly agree/ Agree | | 76 (43%) | |
| I am worried about the long-term effects that COVID-19 will have on my career, personal life, and/or family/friends | not SA/A | | 70 (39%) | |
|  | Strongly agree/ Agree | | 108 (61%) | |
| I am optimistic about the future given the trajectory of the COVID-19 pandemic | Strongly Agree | | 16 (9%) | |
|  | Agree | | 52 (30%) | |
|  | Neither Agree nor Disagree | | 38 (22%) | |
|  | Disagree | | 53 (30%) | |
|  | Strongly Disagree | | 16 (9%) | |
| Stress outcome | Neither Agree nor Disagree | | 2 (1%) | |
|  | No | | 24 (14%) | |
|  | Yes | | 150 (85%) | |
| Policy related stress | Neither Agree nor Disagree | | 9 (5%) | |
|  | No | | 17 (10%) | |
|  | Yes | | 150 (85%) | |
| Productivity outcome | Neither Agree nor Disagree | | 12 (7%) | |
|  | No | | 26 (15%) | |
|  | Yes | | 131 (78%) | |
| Optimism outcome | Neither Agree nor Disagree | | 38 (22%) | |
|  | No | | 69 (39%) | |
|  | Yes | | 68 (39%) | |
