# Supplementary material for: Impact of COVID-19 pandemic on physician-scientist trainees to faculty one year into the pandemic
Source: BMC Med Educ. 2024 May 28;24:587. doi: 10.1186/s12909-024-05541-9 (PMC11134762; doi:10.1186/s12909-024-05541-9)
Supplement: Supplementary file 5 — Supplementary Material 5. [file 12909_2024_5541_MOESM5_ESM.docx]

**Supplemental table 2.** Table of time spent on various activities by respondent cohort.

Childcare/homeschooling

MS: 0: 88.4%, >0: 11.6%

GS: 0: 88.5%, >0: 11.5%

RFJF: 0: 43.2%, >0: 56.8%

Administrative duties

MS: <=45: 81%, >45: 19%

GS: <=45: 99%, >45: 1%

RFJF: <=45: 96%, >45: 4%

Clinical duties

MS: <=45: 57%, >45: 43%

GS: <=45: 97%, >45: 3%

RFJF: <=45: 51.2%, >45: 48.8%

Research/Scholarly Activities

MS: <=45: 57%, >45: 43%

GS: <=45: 18.1% , >45: 81.9%

RFJF: <=45: 64.6%, >45: 35.4%

Personal Time

MS: <=45: 91%, >45: 9%

GS: <=45: 97%, >45: 3%

RFJF: <=45: 99%, >45: 1%

Volunteer Activities

MS: <=45: 91.6%, >45: 8.4%

GS: <=45: 100%, >45: 0%

RFJF: <=45: 100%, >45: 0%
